# On the Lateral Compressive Behavior of Empty and Ex-Situ Aluminum Foam-Filled Tubes at High Temperature

**DOI:** 10.3390/ma11040554

**Published:** 2018-04-04

**Authors:** Emanoil Linul, Nima Movahedi, Liviu Marsavina

**Affiliations:** 1 Department of Mechanics and Strength of Materials, Politehnica University of Timisoara, 1 Mihai Viteazu Avenue, 300 222 Timisoara, Romania; liviu.marsavina@upt.ro; 2 Independent Researcher (Graduated from Department of Metallurgy and Materials Engineering, Semnan University, University Blvd.), 35131-19111 Semnan, Iran

**Keywords:** aluminum foams, ex situ foam filled tubes, compression tests, temperature, energy absorption

## Abstract

In this research work, the effect of lateral loading (LL) on the crushing performance of empty tubes (ETs) and ex situ aluminum foam-filled tubes (FFTs) was investigated at 300 °C. The cylindrical thin-walled steel tube was filled with the closed-cell aluminum alloy foam that compressed under quasi-static loading conditions. During the compression test, the main mechanical properties of the ETs improved due to the interaction effect between the cellular structure of the foam and the inner wall of the empty tube. In addition, the initial propagated cracks on the steel tubes reduced considerably as a result of such interaction. Furthermore, the obtained results of the LL loading were compared with the axial loading (AL) results for both ETs and FFTs at the same temperature. The findings indicated that the application of loading on the lateral surface of the composite causes the lower mechanical properties of both ETs and FFTs in comparison with the axial loading conditions.

## 1. Introduction

Compared to fully-dense solid metals, metallic foams are a new class of ultra-lightweight structural materials that are highly valued in recent years in many crucial engineering fields (such as aircraft, spacecraft, vehicles, and ships), due to their excellent performances in energy absorbing and mechanical damping [1,2,3,4]. Many elements are designed to absorb the impact kinetic energy employing the cellular materials or thin-walled tubes.

There is a broad range of studies carried out on manufacturing processes of metal foams [5,6,7,8], recently developed composite foams [9,10,11,12,13] and their characterization [14,15,16,17,18]. Li et al. [19] presents a comparative analysis of crashworthiness of empty and foam-filled thin walled tubes with various section shapes (empty tubes, foam-filled single tubes, foam-filled double tubes, and corner-foam-filled tubes) under quasi-static axial compression tests. Mechanical response and energy absorption of aluminum foam-filled and empty circular tubes with different geometries were investigated by [20]. The effects of length, outer diameter, wall thickness of specimen, morphology and density of foam on the mechanical response are discussed. Goel [21] carries out deformation and energy absorption studies with single, double, and multi-wall square and circular tube structure with and without aluminum foam cores for assessing its effectiveness in crashworthiness under identical test conditions. Langseth and co-workers [22,23], Gibson and Ashby [24], Duarte et al. [25], and Guillow et al. [26] analyzed the deformation mechanisms and mechanical performances of the foam-filled tubes under axial compressive loading conditions. Ahmad et al. [27] have examined the crush response and energy absorption of empty and foam-filled conical tubes under oblique impact loading.

It was found that the previous experimental studies on the mechanical properties of the empty tubes (ETs) and the aluminum foam-filled tubes (FFTs) were mostly focused on the axial crushing behavior and energy absorption at room temperature, while only a limited number of studies have been performed on their high-temperature mechanical properties. Wang et al. [28] studied the failure mode, crush force efficiency, energy ductility coefficient, and specific energy absorption of foam-filled circular glass fiber-reinforced plastic (GFRP) tube under high temperatures. Their results show that the axial crush behavior and energy absorption capacity of foam-filled GFRP tubes were determined by the testing temperature. Recently, Linul et al. [29] and Movahedi and Linul [30] investigated the axial compression behavior of ETs and FFTs specimens at different temperatures and concluded that increasing the testing temperature directly affects the mechanical properties and stability of the composite structure.

Actually, owing to their wide range of applications, these advanced composite materials are used under different loading conditions (axial and lateral loading) and at different temperatures (low and high operating temperature). In practice, the effects of high temperatures on the composite’s mechanical behavior cannot be ignored because porous (foam) materials are very sensitive to temperature changes [28]. According to author’s knowledge, up to now there are no experimental reports in the literature with respect to the mechanical properties under lateral loading (LL) of FFTs at higher temperatures. Therefore, the aim of this research work would mainly focus on the simultaneous effect of sample position (LL direction) and temperature (300 °C) on the mechanical response of both the ETs and of the ex situ FFTs under quasi-static compression loading. Finally, a comparative study on the crushing behavior of both loading configurations (LL and AL directions) was investigated. The most important reason to select 300 °C in this research is related to the softening of the matrix alloy (which the closed-cell aluminum foam has been made of) at this temperature, which makes the effect of temperature more tangible on the mechanical behavior of the composite.

## 2. Materials and Methods

### 2.1. Materials

**Figure 1:**
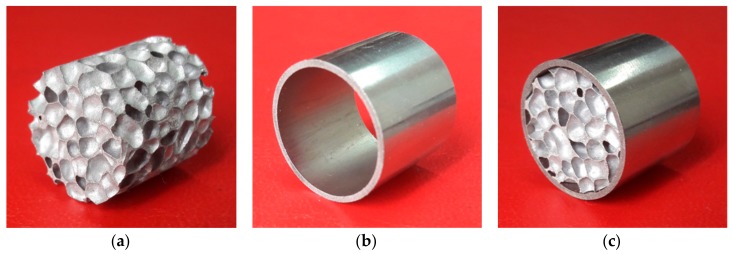
Samples configurations: aluminum foam (**a**); empty tube (**b**); and foam-filled tube (**c**).

Closed-cell aluminum alloy foam (Figure 1a) for this study was produced by casting method. The details of samples preparation have been described in [29]. In addition, in order to investigate the behavior of ex-situ FFTs the 304 stainless steel tube with wall thickness of 1 mm was used (Figure 1b). In this case, the cut cylindrical foam samples were inserted within the steel tubes by a press-fit technique (Figure 1c). 

### 2.2. Methods

The lateral quasi-static compressive loading tests of the samples was carried out using100 kN LBG testing machine with a loading speed of 10 mm/min according to ISO 13314 standard [31]. In order to keep the samples at desired temperature during the test, the compressive testing machine was equipped with a thermal chamber.

## 3. Results and Discussions

**Figure 2:**
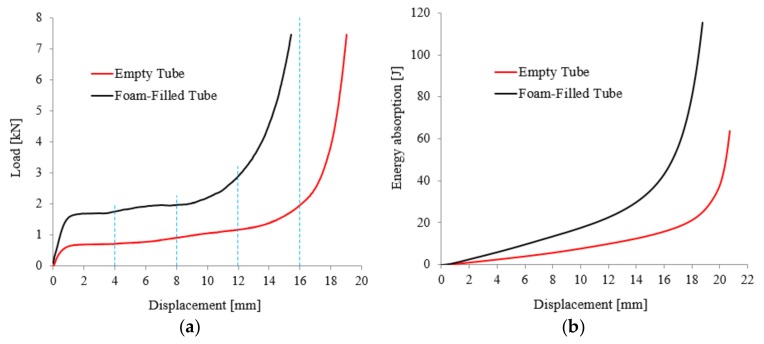
F-Δ curves (**a**) and W-Δ variation (**b**) of ETs and FFTs samples configurations.

Figure 2a presents the load (F)-displacement (Δ) curves of the investigated ETs and FFTs samples at 300 °C. From the obtained F-Δ curves, three different regions were found similar to other foams and composite foam structures [32]: a linear-elastic region up to a displacement value of 0.75 mm (around 3% strain); followed by a plateau region (between 3–50% strain) with a slight increase in load where the most important collapse mechanism has occurred; and, finally, ends with a densification region (after 50% strain) with a significant increase in load. The FFTs yield load (F_y_) capacity is 62% higher than that of the ETs, while both plateau (F_pl_) and densification (F_D_) loads also show higher values up to 54%. On the other hand, the energy absorption (W_D_) values at the onset displacement of densification shows a value of 18.52 J for FFTs compared to ETs that is only 9.58 J (see Figure 2b). The shape of the plateau region represents an important factor in the selection of cellular materials for energy absorption applications. Due to the cell-walls collapse mechanisms, during this stage, the largest amount of energy at an almost constant load occurs [33,34]. 

**Figure 3:**
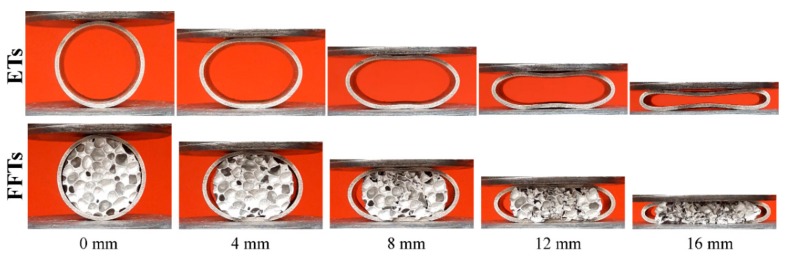
Deformation sequences of ETs and FFTs under LL quasi-static compression loadings.

As can be seen in Figure 3 the thin-walled cylindrical ETs specimen presents a curved surface (between 8–16 mm displacements) under LL condition, while in the case of FFTs samples the non-deviated deformed plane surface of the ETs were observed that would be related to the energy absorption effect of closed-cell aluminum foams. The use of aluminum foam as a core material increases both the strength of the structure and its stability under compressive loads at elevated temperature, even despite the fact that the core foam within the FFTs at the initial stages of compression (4 mm of displacement) starts to be compressed and separate from the inner wall of the steel tube.

**Figure 4:**
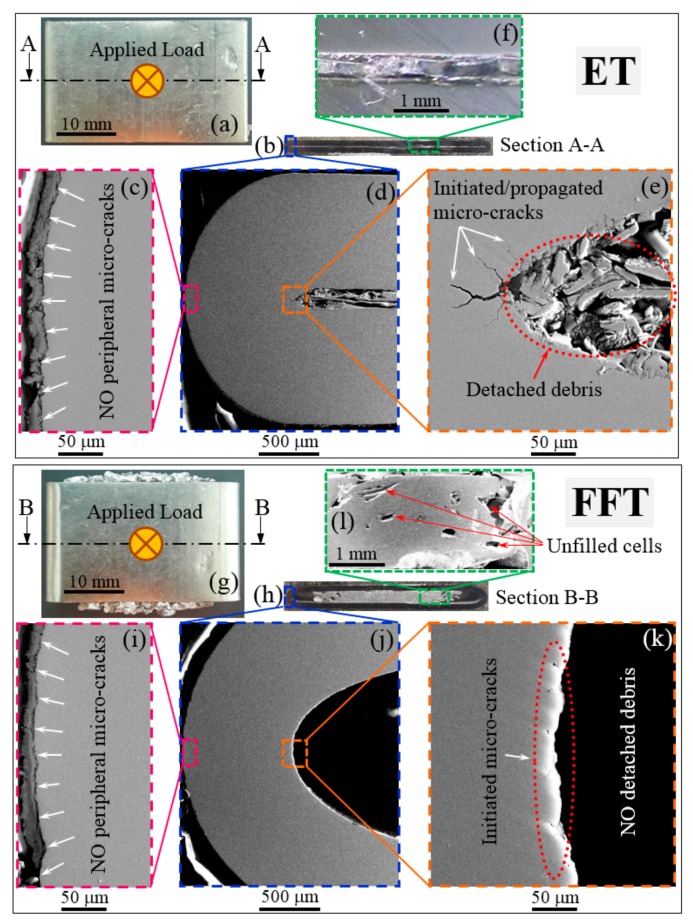
Macroscopic (**a**,**b**,**f**,**g**,**h**,**l**) and microscopic (**c**–**e**,**i**–**k**) images of initiated and propagated micro-cracks in ETs and FFTs specimens.

Figure 4b,h presents the macrostructural images of the cross-section of the investigated samples. To achieve a more accurate comparison between the two structures (ET and FFT), tested samples were loaded with a force having the same magnitude, in this case 60 kN (Figure 4a,g). The authors consider that comparing the response of both empty and foam filled tubes to a given load (as opposed to comparing the response at a given deformation) is more relevant in determining the load bearing capabilities of the composite structure. As shown in SEM images from Figure 4c,i, there are no visible peripheral cracks in the outside area of the deformed ET and FFT, only a slight plastic deformation of the steel tube that might be related to the simultaneous effect of testing temperature and compressive loading. In the inner part of the ET (Figure 4e), many micro-cracks (up to 100 µm length) have been initiated and propagated in the wall of the steel tube. Furthermore, in the inner part of the tube, considerable parts of the tube material began to detach that might play as the stress concentration centers in adjacent to the inner wall surface of the steel tube. This debris is one of the factors that influences the deterioration of the mechanical properties of the empty steel tube, as well as the lack of a foam core (see Figure 2a,b), leading, ultimately, to an unstable collapse of the empty structure.

On the other hand, the use of Al foam as a filler material greatly decreases the initiation and propagation of the micro-cracks in the steel tube (Figure 4j). In this case, there is no detached debris from the inner part of the tube wall (Figure 4k). This deformation mechanism is due to the closed cellular structure of the aluminum foam, which stabilizes the collapse mode of the composite structure. It can be seen that the inner walls of the FFT specimen are not in contact with each other (see Figure 4h). They, together with a great deal of unfilled Al foam cells (Figure 4l), can still withstand even higher loads than 60 kN, resulting in the absorption of a much larger amount of energy. On the opposite side, the inside walls of the ET specimen are in contact at the mentioned load (see Figure 4b,f), absorbing an energy twice lower than the FFT specimen. 

**Figure 5:**
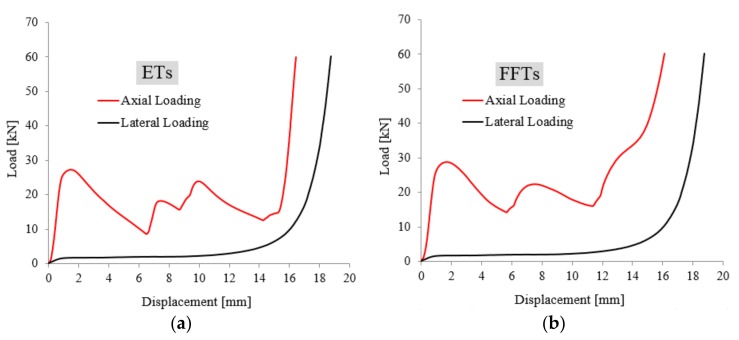
A comparison of axial and lateral load-displacement curves for both ETs (**a**) and FFTs (**b**) sample configurations.

According to [29] five different regions were observed for load-displacement curves under the AL direction with a high level of load oscillations during elevated compression tests, while only three regions for LL direction. From Figure 5a,b it can be easily observed that both sample configurations (ETs and FFTs samples) exhibit much lower mechanical properties under lateral compression loads than in the case of axial ones, which might be related to the lower loading surface and the anisotropy effect of the sample in the lateral direction. The typical load-displacement curves morphology (with different numbers of regions and different oscillations) and the major difference between the values of the mechanical properties under the two load conditions are given by the much smaller loading area for LL direction (44 mm^2^) compared to AL direction (379.94 mm^2^). 

**Figure 6:**
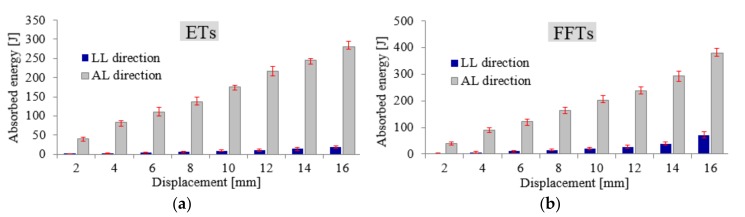
A comparison of axial and lateral energy absorption values for ETs (**a**) and FFTs (**b**) sample configurations.

A comparison between two investigated loading directions of FFTs shows that, under LL direction (see Figure 6b), the energy absorption reduction (W_R_) was about 93% at low displacements (<5 mm), and it decreases polynomially up to a value of 81% for large displacements (>15 mm). 

In the case of ETs specimens (see Figure 6a), the W reduction was even greater, reaching a value of 97% for low displacements and 93% for larger displacements. From analyzing Figure 6, it seems that the different positioning of cylindrical samples under the compressive loading device significantly influences the load bearing capacity of the investigated structure.

## 4. Conclusions

This paper, for the first time, experimentally investigates the effect of the LL direction on the compressive crushing behavior of cylindrical thin-walled ETs and ex situ FFTs at elevated temperature (300 °C). The investigation shows that the main mechanical properties of the closed-cell aluminum alloy foam-filled tubes are higher than that of the corresponding thin-walled steel tubes (up to 74% in terms of W and 62% for F_y_). Moreover, due to the interaction effect between the aluminum foams and the ETs, the foam enhances the tube tolerance against deformation in a much more stable manner, without a large number of micro-cracks. It should be highlighted that only the inside wall of the ET specimen presents a high number of propagated micro-cracks while in the case of FFT, such cracks were not visible. The FFT strength is not only a summation of both the mechanical responses of the ET and the foam, but also an additional coupling contribution is obtained due to the interaction effect between the foam and the ET. 

On the other hand, it was shown that the mechanical properties are significantly affected by the loading direction. Therefore, under AL direction, the absorbed energy is higher than that obtained for LL direction (up to 33 times for ETs and 14 times for FFTs).
